# Indicators for National and Global Monitoring of Girls' Menstrual Health and Hygiene: Development of a Priority Shortlist

**DOI:** 10.1016/j.jadohealth.2023.07.017

**Published:** 2023-12

**Authors:** Julie Hennegan, Bethany A. Caruso, Garazi Zulaika, Belen Torondel, Jacquelyn Haver, Penelope A. Phillips-Howard, Jonathan Valdez, Caitlin Gruer, Neville Okwaro, Therese Mahon, Marni Sommer

**Affiliations:** a Maternal, Child and Adolescent Health Program, Burnet Institute, Melbourne, Victoria, Australia; b Melbourne School of Population and Global Health, University of Melbourne, Melbourne, Victoria, Australia; c Hubert Department of Global Health, Rollins School of Public Health, Emory University, Atlanta, Georgia; d Department of Clinical Sciences, Liverpool School of Tropical Medicine, Liverpool, United Kingdom; e Department of Disease Control, London School of Hygiene and Tropical Medicine, London, United Kingdom; f Department of Education and Child Protection, School Health and Nutrition Program, Save the Children US, Washington, D.C.; g Department of Sociomedical Sciences, Mailman School of Public Health, Columbia University, New York, New York; h Ministry of Health, Nairobi, Kenya, Nairobi, Kenya; i International Programmes Department, WaterAid, London, United Kingdom

**Keywords:** Monitoring, WASH, Education, Gender, Menstruation, Adolescent girls, Menstrual health, Measurement, Health policy

## Abstract

**Purpose:**

Despite the importance of menstrual health and hygiene (MHH) for adolescent girls' health, education, and gender equality, few countries monitor MHH. MHH needs remain underprioritized, and progress achieved through policies, programs, or investments go unmeasured. This article reports the systematic development of an indicator shortlist to monitor adolescent girls' MHH at the national and global levels across low- and middle-income countries.

**Methods:**

A core group of MHH researchers and practitioners collaborated with stakeholders from three countries with demonstrated commitment to monitoring MHH (Bangladesh, Kenya, and the Philippines), measures experts, and a global advisory group. The approach included the following: (1) define domains for monitoring MHH; (2) review and map existing indicators and measures; (3) iteratively shortlist indicators through appraising quality, feasibility, and stakeholder input; and (4) refine the shortlist and develop guidance for use.

**Results:**

The shortlist comprises 21 indicators across seven domains covering menstrual materials, water, sanitation, and hygiene facilities, knowledge, discomforts and disorders, supportive social environments, menstrual health impacts, and policies. Indicators are accompanied by measures that have been tested or are expected to provide reliable data, alongside justification for their selection and guidance for use.

**Discussion:**

The shortlisted indicators reflect the multisectoral collaboration necessary for ensuring girls' MHH. Uptake requires integration into monitoring systems at national and global levels. Future work remains to evaluate the performance of the indicators over time and to support their widespread use. Governments and stakeholders can use these indicators to track the progress of programs and policies, monitor unmet MHH needs, identify disparities, and set targets for improvement.

**Implications and Contribution**

A lack of standardized indicators curtails efforts to set and assess progress against targets that support menstrual health and hygiene (MHH), unify approaches, and hold governments and service providers to account. The priority indicators for monitoring MHH for adolescent girls provide governments with guidance for initiating action and enabling comparability.

Adolescent MHH is essential to achieving the sustainable development goals, including gender equality [1,2]. Sustained advocacy over the past decade has seen the importance of MHH recognized, and efforts to support MHH have expanded [[3], [4], [5], [6], [7], [8], [9], [10], [11]]. National-level attention has been varied. In some countries, policies have been comprehensive, addressing multiple MHH needs [12,13]. In others, efforts have been more narrow in scope, for example, through specific campaigns to reduce taxes placed on menstrual products [[14], [15], [16], [17]]. Further, in many countries, little to no policy effort has addressed MHH [7,18,19]. Motivating broader policy attention and monitoring progress requires data capturing MHH circumstances. A lack of data renders girls' MHH needs invisible and curtails necessary investment [19], while a lack of consensus on indicators for monitoring MHH hinders efforts to improve data availability. Improved data and monitoring mechanisms to capture MHH circumstances and track the progress resulting from investments are needed [6,20,21].

Adolescence brings with it the onset of MHH needs and presents a window of opportunity to provide timely menstrual knowledge, support self-care practices, encourage positive interactions with health-care services, and safeguard education. While MHH is relevant to adolescent girls, adult women, and all other people who experience a menstrual cycle during their life course, existing evidence, measures, and policy initiatives have largely concentrated on adolescents. Indicators and measures are thus needed for this life stage to elucidate the prevalence of unmet needs and monitor progress. Our focus reflects (1) the ability to propose evidence-based indicators for adolescents, (2) the need to start tracking policies and programs that do exist, and (3) an urgent need to extend programs and develop indicators/measures for other groups.

Previous efforts to monitor MHH needs have been integrated into surveys or leveraged existing data aligned with specific sectors, providing useful information but an incomplete picture. For example, initial work to estimate the availability of facilities for menstrual management used water, sanitation, and hygiene (WASH) monitoring data reporting the coverage of improved sanitation infrastructure [22], a designation based on a facility's ability to sequester feces [23]. Such indicators did not capture quality or suitability for menstrual tasks [24]. The Performance Monitoring for Action (PMA) survey program across 12 countries collected representative data specific to MHH among women aged 15–49 [25]. PMA surveys captured menstrual product use, facilities used for MHH, self-reported unmet needs, and school and work absenteeism due to menstruation [26,27], delivering initial estimates of some MHH needs and providing opportunities to evaluate measure performance [28,29]. The Joint Monitoring Program of the World Health Organization and United Nation's Children's Fund (UNICEF) incorporated similar questions to the PMA surveys into Multiple Indicator Cluster Surveys. The Joint Monitoring Program’s 2021 progress report included a chapter on menstrual health, with data from 42 countries on at least one of the four indicators they prioritized, including awareness of menstruation at menarche, the use of menstrual absorbents, access to a private space to wash and change, and participation in activities during menstruation [30]. While the data from these efforts have been insightful, the indicators used neither represent a full suite of MHH inputs, outcomes, and impacts worthy of tracking nor were they identified using a systematic approach. A more comprehensive set of indicators—informed by inputs from multiple and diverse actors working in MHH—that builds on these foundational efforts is needed.

Few countries have collected national monitoring data on MHH. In 2014 and 2018, the Bangladesh National Hygiene Surveys captured a range of information about MHH needs, while in the Philippines, the yearly WASH in schools’ online monitoring system captures the presence of school-level facilities, curriculum, financing, and advocacy to support MHH. In Kenya, MHH indicators have been listed for inclusion in school monitoring systems but have not progressed to routine monitoring. India's National Family Health Survey rounds 4 (2015–2016) and 5 (2019–2021) collected national-level data on the use of menstrual absorbents and menstrual cycle awareness [31,32]. These efforts have provided valuable data to assess MHH circumstances nationally, all using different measures and assessing different MHH needs. Comparable data across countries would enable tracking of MHH progress globally and over time, creating greater opportunities for countries to learn from their own program and policy investments and from one another. Comparable measures would also allow the interpretation of monitoring data to be informed by research studies testing indicators and measuring performance or evaluating causal relationships between the indicators and other outcomes of interest.

To support improved, comparable tracking of progress toward girls' MHH in low- and middle-income country (LMIC) contexts nationally and globally, we aimed to develop a shortlist of indicators with accompanying measures for integration into national monitoring efforts.

## Methods

### Approach and collaboration structure

Our development of a shortlist of indicators to monitor MHH built on a preceding effort to identify priority indicators in key development sectors (education, gender, psychosocial health, sexual and reproductive health, and WASH) with a hypothesized link to menstruation [20]. The “Monitoring MHH” work assembled a group of measures and MHH experts for a three-day meeting in 2019 including participants representing each of the core sectors and working in research, government, United Nations-agencies, and Non-Governmental Organizations programming, with advanced input via online survey from a larger global advisory group (see [20] for membership list). The meeting identified the need for a consolidated and comprehensive set of national indicators for MHH.

**Figure 1:**
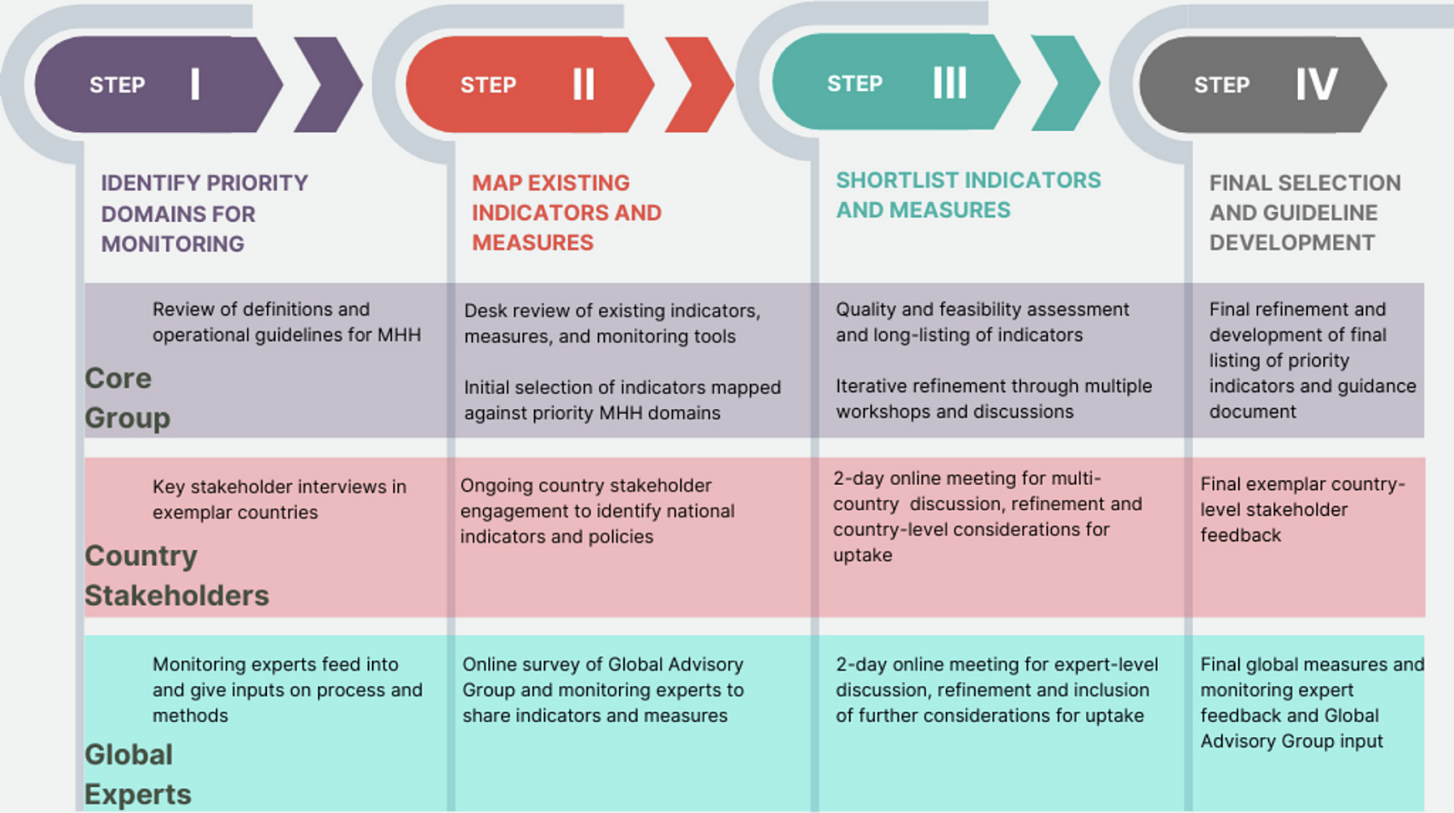
The overlapping four-step process that was undertaken to identify the shortlist of priority indicators for MHH.

Our present effort was undertaken from January 2021 to March 2022 and followed an overlapping four-step process presented in Figure 1, including the (1) identification of priority domains for monitoring, (2) reviewing and mapping of indicators, (3) iterative shortlisting and appraisal, and (4) final shortlist and guidance development. The indicator selection process was designed to balance monitoring the breadth of MHH considerations with the need for a short, feasible, and evidence-based list. Holistic attention to MHH was attended to in step 1 by identifying the breadth of domains reflected in current conceptualizations of MHH. This included incorporating existing definitions and understanding current policy and country stakeholder areas of focus. At least one indicator was identified for each domain. In step 2, candidate indicators were drawn from those that had been used in national or large-scale monitoring or had a significant evidence base, constraining the available indicator set to those with demonstrated feasibility. Step 3 further reduced the set of indicators by prioritizing stakeholder ratings on relevance, feasibility (including where data were already being collected), and usefulness. These ratings were expanded upon in a two-day meeting of expert stakeholders to discuss feedback and suggest revisions to indicators. There was no *a priori* limit to the number of indicators that could be included in the list; however, stakeholder feedback emphasized brevity.

The work was led by a core group of MHH researchers and practitioners with members from the preceding measures meeting, and the MHH group and global advisory group were consulted throughout. Formal engagement with representatives from government departments engaged in MHH policy, program implementation, or monitoring in three exemplar countries (Bangladesh, Kenya, and the Philippines) fed into each step. To select exemplar countries, first, countries were identified that had demonstrated government-led prioritization of MHH and commitment toward national-level monitoring of MHH. This was essential to ground the indicator shortlist in country experience and priorities. Second, countries were selected where core group members had experience working with government stakeholders to ensure feasibility. These connections were more important as the project was implemented during the COVID-19 pandemic, and engagement became virtual. Select inputs were also captured from the government of South Africa, given its expanding commitment to MHH.

### Step I: Identifying priority domains for monitoring

The conceptualization of MHH has evolved rapidly. MHH is relevant to a range of sectors and closely linked with multiple sustainable development goals [1]. The core group drew on definitions of “menstrual hygiene management,” [33,34] “menstrual health and hygiene,” [35,36] and “menstrual health,” [37] along with operational pillars for MHH outlined by UNICEF's guidance for MHH [35] and the menstrual hygiene management in Emergencies Toolkit [38]. We extracted domains of MHH by thematically grouping concepts across these conceptualizations. The MHH needs domains reflect a synthesis of all the thematic concepts identified within extant conceptualizations, in addition to one domain representing the impacts of MHH on girls' lives. One higher-level domain, the policy context, was added to reflect this unique level of monitoring sitting across topic areas. These domains outline the range of concepts that require monitoring to provide a holistic picture of girls' MHH.

**Figure 2:**
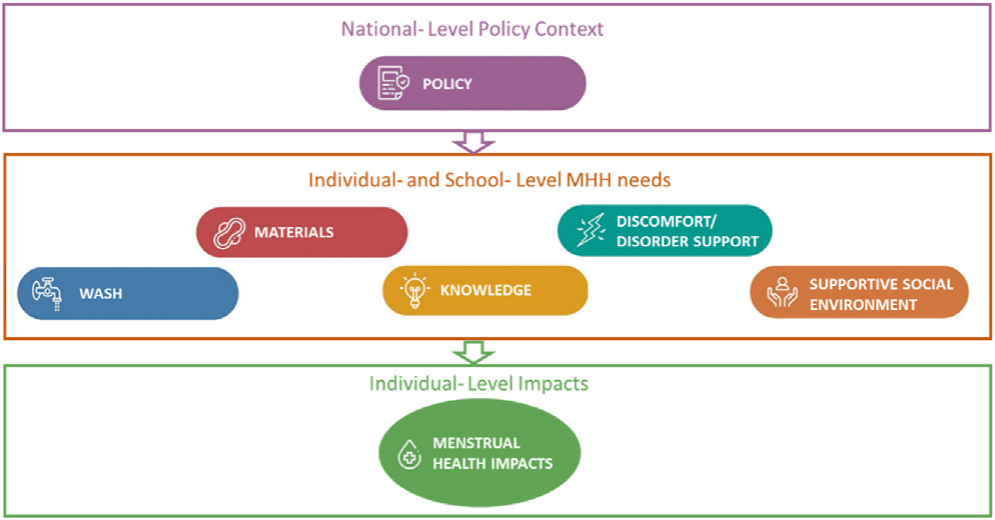
The seven priority domains for monitoring adolescent girls' menstrual health and hygiene including five capturing the requirements needed for achieving MHH (materials, facilities, knowledge, support for discomforts and disorders, and a supportive social environment), in addition to a domain capturing menstrual health impacts and a domain monitoring the policy context.

Seven priority domains for monitoring MHH were identified, as presented in Figure 2.

### Step II: Concomitant review and mapping indicators and measures

#### Exemplar country engagement

Following an inception meeting in each exemplar country to describe the project, we undertook informational interviews with representatives from relevant ministries encompassing WASH, environment, health, gender, population, and education. Interviews sought to (1) understand MHH-related priorities and policy and program efforts, (2) identify existing indicators or monitoring efforts related to MHH, (3) explore current data use, (4) identify perceived challenges and enablers to indicator implementation and data use, and (5) seek recommendations for shortlist dissemination. These interviews were to capture insights into monitoring approaches and thus did not require seeking ethics committee approval for human subjects’ research.

Eighteen individual and small group interviews were undertaken (9 in Bangladesh, three in the Philippines, and six in Kenya), alongside requests for documentation and exchanges via email. Insights into challenges, enablers, and uptake were summarized in presentations viewed asynchronously ahead of a two-day online meeting conducted in June 2021. Country stakeholders reviewed presentations and joined the online meeting to share lessons learned and provide feedback on the draft indicator shortlist. Debriefing meetings were held with stakeholders following the consultation.

#### Desk review

We undertook a desk review to identify existing indicators and measures. Eligible indicators and measures were those that had previously been collected at nationally or subnationally representative levels, in large-scale surveys, included in policy documentation, or in measure development studies. We also reviewed measures embedded within rigorous studies such as randomized trials and relevant MHH summary reports, such as the UNICEF guidance on monitoring MHH [39], to ensure potential validated measures were not missed. Measures had to be publicly available or shared with the core group upon request.

Indicators and measures were identified through searches in multiple academic databases, along with Google searches for gray literature, policy, or monitoring tools shared through engagement with exemplar countries, and an online survey of the global advisory group which asked members to share (1) indicators they believed should be considered for a shortlist; (2) relevant tools, toolkits, or measures that should be included in the desk review for consideration; and (3) relevant data sources.

Our review built on efforts by members of the core group who had undertaken reviews of policy attention to MHH [18], the measurement of MHH in research studies [40], and the development of indicators for gender-sensitive WASH monitoring [[41], [42], [43]]. Further, core group members had contributed to UNICEF's 2020 guidelines for monitoring MHH in programming [39], and the collation of MHH measures aggregated in the development of the guidance was shared with the core group to support the review.

#### Mapping indicators and measures

A total of 154 indicators and related measures were identified by the initial review as candidates for national monitoring. Country engagement and desk review found little consistency in the indicators used across countries. Consultations indicated that limited national monitoring had been undertaken, and validation or testing had rarely been conducted.

A spreadsheet was developed that displayed the indicators collected from the review, the indicator level of measurement (individual, school, other), the questions or measures used to populate the indicator, efforts in which the indicator or measure had been used, and a summary of any validation efforts related to the measure. Subsets of the spreadsheet were developed to extract indicators relevant to different development sectors and those indicators that had been used in each of the three exemplar countries to facilitate familiarization and comparison.

### Step III: Iterative shortlisting through appraisal of quality and feasibility

To map the identified indicators according to priority domains (Figure 2), develop subgroups of indicators within each domain, and identify gaps in the available indicators, the core group undertook iterative rounds of asynchronous reviews followed by online meetings led by an external facilitator.

Where multiple indicators were identified assessing similar concepts, a single indicator was selected. For example, many similar questions capturing knowledge about the menstrual cycle were identified, and so the core group selected two indicators from this larger set, *awareness of menstruation prior to menarche* which had been successfully used in national monitoring efforts [30,44] and *knowledge of the fertile window* as this is used in Demographic and Household Surveys [45]. Where feasible, the core group developed indicators and measures for identified gaps or refined measures for identified indicators based on the available evidence base and critiques of measure validity. A total of 38 indicators remained after initial mapping. The core group then undertook an initial assessment of indicator quality and feasibility using a three-level rating system (see Supplementary Materials).

This work developed an initial set of 27 indicators and measures that were shared with the global advisory group for feedback. Inputs were compiled against the indicators and reviewed by the core group, which informed further refinement.

To formally appraise the candidate indicators, the core group, measures, and MHH expert group undertook a week of asynchronous online review. A total of 17 indicators, including some groupings of indicators that were rated together, were loaded into the “PowerNoodle” online web platform [46]. Participants were invited to rate indicators on relevance, usefulness, and feasibility (definitions reported in Supplementary Materials). PowerNoodle also allowed participants to comment on each indicator. Comments showed publicly in the system, and participants were reminded to return to the site and respond to the comments posted by others.

Asynchronous ratings and comments informed a two-day online meeting during which breakout groups dedicated to each domain area were assigned for in-depth discussion to make notes on suggested refinement, missing indicators, and considerations on inclusion of indicators in the shortlist. A summary of reflections was then shared during plenary sessions. The second day of the meeting also included country stakeholder feedback on feasibility, reflection on the process, and focused consultation on strategies for dissemination and uptake.

### Step IV: Final shortlist and guidance development

Repeated meetings of the core group integrated feedback from the asynchronous review and two-day meeting. Indicators and measures were selected for a draft shortlist and further refined. This list was shared again with the measures and MHH expert group, global advisory group, and country experts for final feedback. After integrating final feedback, the core group developed guidance for the shortlist, and a guidance report was released in March 2022 [47] followed by a series of webinars for dissemination.

## Results

The shortlist of recommended indicators for monitoring MHH at national and global levels is presented in Table 1. Twenty-one indicators were selected, including three policy input indicators, seven school-level output indicators, nine individual-level outcome indicators, and two impact indicators assessed at the individual level. Table 1 also presents brief justification for each indicator and evidence relevant to the recommended measure. Detailed guidance for the use of each indicator and the associated measures and considerations for use are outlined in the technical guidance report [47].

**Table 1:** Final shortlist of indicators for monitoring adolescent girls' menstrual health and hygiene at national and global level

Domain	Indicator	Evidence and importance	Indicator type
Materials	1. % of girls who reported having enough menstrual materials during their last period.	A single individual-level indicator was selected to capture girls' access to materials to catch or absorb menses with sufficient clean material, an essential requirement for MHH [33,37]. This indicator was developed based on an item from the Menstrual Practice Needs Scale [48] to provide an assessment of access to sufficient quantities of absorbent. This was prioritized as a basic level of assessment, where use of preferred materials could be considered indicative of greater access.	Individual outcome
Materials	2. % of schools with menstrual materials available to girls in case of an emergency.	The availability of materials in schools in the case of unexpected need was a new indicator developed by the core group adapted from data collected in the Philippines Department of Education monitoring [49]. The indicator reflects policies aimed at supporting consistent access to menstrual materials. It does not require the availability of free products for all of girls' material needs as this approach is pursued in a limited number of countries and may not be feasible or best practice for all.	School output
WASH	3. % of girls who reported changing their menstrual materials during their last menstrual period at school.	MHH requires supportive facilities to care for the body. The experience of these spaces can only be captured among those using them by capturing the proportion of girls who have used school spaces for changing menstrual materials. Indicator 3 provides a denominator for indicator 4, along with context for interpreting indicators 5–7 as the presence and quality of facilities are relevant if they are being used.	Individual outcome
WASH	4. % of girls who changed their menstrual materials at school in a space that was clean, private, and safe during their last menstrual period.	Indicator 4 is an individual-level indicator capturing girls' experience of menstrual-friendly facilities for blood management tasks at school. Nationally representative data on the quality (cleanliness, privacy, safety) of spaces used to change menstrual materials have been collected in PMA [25] and JMP MICS [30] programs, with the shortlisted indicator drawing on advancements in assessing privacy and safety in self-report [29,48,50].	Individual outcome
WASH	5. % of schools (primary/secondary) with improved sanitation facilities that are single-sex and usable (available, functional, and private) at the time of the survey.	Indicators 5–7 can be assessed at the school level to appraise the quality of facilities provided to girls for menstrual self-care. Increasing criteria are applied, capturing the availability of facilities, menstrual-friendly features, and the availability of resources for washing. Indicators are drawn from monitoring undertaken by the JMP [51] and in two exemplar countries (Bangladesh and the Philippines) [49,52]. The indicator provides direct feedback to government on the condition of supportive WASH facilities in schools for MHH.	School output
WASH	6. % of (primary/secondary) schools with improved sanitation facilities that are single-sex, usable (available, functional, and private), lockable from the inside, have covered disposal bins, and have disposal mechanisms at the time of the survey.		School output
WASH	7. % of (primary/secondary) schools that have water and soap available in a private space for girls to manage menstruation.		School output
Knowledge	8. % of students (male/female) who have received education about menstruation in primary and secondary school.	An individual-level outcome monitored in exemplar country, Bangladesh [52], this indicator captures the receipt of menstrual education. The indicator can be captured for male and female students. As a basic level of assessment, the indicator captures the receipt of any education, with additional criteria such as quality or content feasible for higher-level assessments.	Individual outcome
Knowledge	9. % of females who know about menstruation prior to menarche.	Timely menstrual cycle knowledge is essential for MHH. Awareness of menstruation at menarche has been assessed in nationally representative surveys and many smaller data collections [30,44,53]. It was developed as an indicator and recommended by the 2019 Monitoring MHH activities preceding the shortlisting effort [20].	Individual outcome
Knowledge	10. % of females with correct knowledge of the fertile period during the ovulatory cycle.	Indicator 10 has been routinely captured for women 15–49 in nationally representative Demographic and Health Surveys [45], assessing key knowledge related to the menstrual cycle and its links with reproductive health and fertility [32]. Thus, data are available for older age groups, and the indicator can be incorporated into assessments among adolescent girls to provide a more comprehensive picture of menstrual cycle awareness and education received.	Individual outcome
Knowledge	11. % of schools where education about menstruation is provided for students from age 9.	Indicator 11 captures school-level implementation of menstrual education in curriculum, providing direct feedback where policies or plans incorporate attention to menstruation in curriculum or a baseline where the guidance is absence. The indicator is drawn from monitoring in two exemplar countries (Bangladesh and the Philippines) [49,52]. Education prior to age 9 was selected to target information provision prior to menarche.	School output
Knowledge	12. Existence of preservice or in-service teacher training about menstruation at the primary or secondary level.	The quality of menstrual education provided to girls is shaped by the support and training provided to teachers. Ensuring teacher comfort with menstrual topics also enables a supportive environment for menstruation in schools. Indicators 12 and 13 capture the coverage of training to equip teachers to provide education about menstruation. The indicator is based on current national monitoring in the Philippines [49] and could facilitate country-relevant targets being set, for example, a proportion of teachers beyond “at least one.”	School output
Knowledge	13. % of schools that have at least one teacher trained to educate primary/secondary students about menstruation.		School output
Knowledge	14. % of countries where national policy mandates education about menstruation at primary and secondary level.	This indicator is for use in global monitoring and was developed during the shortlisting activities to monitor the status of education mandated for menstruation across countries.	Policy input
Discomfort/disorders	15. % of girls who report that they were able to reduce their menstrual (abdominal/back/cramping) pain when they needed to during their last menstrual period.	Review of current indicators and monitoring indicated gaps in available measures related to pain management or care for menstrual discomforts and disorders. Two individual indicators were developed by the core group. Indicator 15 provides a basic indication of girls' ability to reduce pain, informed by recent measures [54]; it does not provide information on the degree of relief or strategies for pain relief.	Individual outcome
Discomfort/disorders	16. % of girls who would feel comfortable seeking help for menstrual problems from a health-care provider.	Menstrual concerns may require treatment or support from health-care providers. Indicator 16 was also developed by the core group to provide an indication of comfort approaching care providers, as a minimal step required for providing adolescent-friendly care for menstrual health concerns.	Individual outcome
Supportive social environment	17. % of girls who have someone they feel comfortable asking for support (advice, resources, emotional support) regarding menstruation.	MHH requires a supportive environment, and adolescents require access to courses of advice and support. Review of indicators identified a dearth of evidence-based measures capturing psychosocial concepts. Indicator 17 was developed by the core group, drawn from measures used in research studies seeking to capture support for MHH [55,56].	Individual outcome
Menstrual health impacts	18. % of girls who report a period does not impact their day.	While most indicators capture requirements for supporting menstrual health, two indicators were identified to capture impacts on adolescent girls' lives. Indicator 18 was derived from the Global Early Adolescence Study, and similar measures used in research studies, to assess any overarching impact of menstruation on daily life [[57], [58], [59]].	Individual impact
Menstrual health impacts	19. % of girls whose class participation was not impacted by their last period.	Indicator 19 was developed based on indicators used in PMA and JMP MICS programs focused on the impacts of MHH on attendance at school or work [26,30]. The indicator was modified to capture the influence on participation, with absenteeism due to menstruation often difficult to capture accurately or sensitive to girls' discomforts or challenges while attending school [60,61].	Individual impact
Policy	20. % of countries with policies or plans that include menstrual health and hygiene.	Indicators 20 and 21 support global monitoring of progress toward supporting MHH. Informed by the GLAAS survey [62], these indicators capture the presence of policies or plans including MHH, such as those developed in the exemplar country, Kenya [12]. The importance of budget allocation was emphasized by exemplar country experts and is assessed in indicator 21, building on the World Bank SABER School Health Questionnaire [63].	Policy input
Policy	21. National budget is allocated to menstrual health and hygiene; funds are dispersed to the schools in a timely and efficient manner.		Policy input

GLAAS, global analysis and assessment of sanitation and drinking-water; JMP = Joint Monitoring Program; MHH = menstrual health and hygiene; MICS = Multiple Indicator Cluster Surveys; SABER, systems approach for better education results; WASH = water, sanitation, and hygiene.

## Discussion

We developed a priority shortlist of indicators to monitor girls' MHH in LMIC settings through a multistep approach, drawing on existing monitoring efforts, evidence, and engagement with exemplar countries. The shortlist provides a consolidated indicator set to address the absence of data reporting MHH circumstances and track progress. The shortlist sought to balance capturing the breadth of domains relevant to MHH with the availability of evidenced and feasible indicators for each domain. The list provides a starting point for improved monitoring nationally and globally.

Moving beyond the limitations of sector-specific indicator sets, we identified seven priority domains for monitoring MHH. Priority domains capture essential requirements for MHH, including timely menstrual knowledge, access to materials and facilities for blood management, care for discomforts and disorders, and a supportive social environment [33,36,37]. In addition, domains assess the impacts of MHH on girls' lives and the policy context. Selecting indicators for these domains was supported through engagement with diverse stakeholders. In exemplar country consultation, this meant engagement with multiple government ministries. Use of the indicators and their measures to assess MHH across the domains at national level will require ongoing national government interagency collaboration and cross-sectoral engagement. Such cooperation is required to support holistic MHH and may serve as a model for other cross-cutting health challenges [64,65]. The cross-sectoral nature of the indicators also presents challenges for uptake and use. Indicators may be best collected by different national monitoring systems (in different sectors or departments) and will require cross-sectoral mechanisms to consolidate data collected for reporting at national or global level.

The shortlist includes indicators measured at different levels, including policy input, institutional output, and individual outcomes. Monitoring across the results chain enables triangulation to provide a more accurate assessment of national MHH status, tracking progress made in both the resources and support being provided and the resulting changes in girls' own experiences. Further, in the guidance document, we recommend collecting demographic data to understand if and how indicators vary by various characteristics. Performing analyses that stratify by age, grade, race, ethnicity, disability, gender identity, or other social identifiers can also identify inequities in the population and therefore signpost where or to whom efforts should be targeted.

To support the feasibility of uptake and to build on the best available evidence, the shortlist prioritized indicators already collected at a national scale with many indicators drawn from monitoring efforts underway in the exemplar countries [49,52]. However, our review found that there were few established indicators for some priority domains. Long-term leadership from the WASH sector meant more indicators related to menstrual blood management had been collected at the national level [25,30] and had opportunities for critique or quality appraisal (e.g., [29,66]). In contrast, indicators capturing support for menstrual discomforts and disorders, supportive social environments, or menstrual health impacts had few available measures. Thus, there is greater granularity provided for some identified domains than others. Methods for assessing psychosocial constructs specific to menstrual health research such as stigma related to menstruation are highly limited [40]. Moreover, national-level monitoring requires short, often single-item, measures. The measurement of latent constructs such as menstrual stigma typically require multiitem scales which are likely to be infeasible in national assessments and require multiple levels of testing for validity and reliability with no measures with sufficient evidence available at the time of shortlist development [67]. Future research evaluating the predictive validity of all suggested indicators in the shortlist and the development and validation of new potential indicators, such as for psychosocial constructs, would support interpreting national monitoring data and the refinement of future indicators.

Most research and monitoring for MHH have focused on adolescent girls. More research is needed to identify appropriate indicators for monitoring MHH experiences across the life course, which can be developed from the work presented here; initial efforts to identify and pilot MHH indicators for those who work outside the home have already leveraged the approaches and learning from this effort [68]. Similarly, monitoring for individual MHH programs may be informed by the indicator shortlist for national monitoring but is likely to require additional indicators, and more extensive measures will be required in MHH research studies. The shortlist presented here draws on evidence, monitoring, and policy in LMIC contexts. While the list may serve as a starting point for monitoring in high-income country settings, some adaptation for these settings is likely to be needed.

Many of the shortlisted indicators capture a minimum level of information on MHH, providing an opportunity to assess needs prior to establishing national programs. The indicators are not positioned to capture all optimal MHH circumstances. For example, indicator nine captures the proportion of adolescent girls with awareness of menstruation prior to menarche. This represents the lowest level of possible knowledge required for this experience [44]. Similarly, indicator 16 assesses the proportion of adolescent girls who report feeling comfortable to seek care for menstrual problems but does not assess care seeking or the quality of care provision.

Development of this shortlist for national and global monitoring represents an important step in ensuring MHH is visible and prioritized. Many more steps are needed to ensure uptake, rigorous data collection, aggregations, and reporting. The indicator shortlist provides a resource for countries to select high-priority indicators for integration into national monitoring when opportunities arise, such as when updating health monitoring information systems. A national infrastructure is required to nest the suggested indicators and may require further training to administer the defined measures. Systems for reporting and using the data will be required with budget lines to support ongoing monitoring and decision-making to ensure monitored data will inform policy, implementation, and best practice. Global bodies with mandates to support monitoring efforts may also identify opportunities to integrate the recommended indicators into their reports and provide frameworks for data reporting over time. Future investments are needed to test and strengthen the indicators, with complementary MHH research to inform evidence-based interpretation of monitoring findings and policy responses.

## Data Availability Statement

No data are associated with this article.
