# Supplementary material for: Indicators for National and Global Monitoring of Girls' Menstrual Health and Hygiene: Development of a Priority Shortlist
Source: J Adolesc Health. 2023 Dec;73(6):992–1001. doi: 10.1016/j.jadohealth.2023.07.017 (PMC10654045; doi:10.1016/j.jadohealth.2023.07.017)
Supplement: Supplementary Materials 1 [file mmc1.docx]

**Supplementary Materials 1**

**Core group initial 3-level rating system**

Indicators and measures were rated on quality and feasibility using a red-orange-green system.

| Green | Recommended candidate for shortlist: few concerns related to quality or feasibility |
| --- | --- |
| Orange | Possible candidate for shortlist: some concerns related to feasibility or quality |
| Red | Exclude from further consideration: significant concerns related to feasibility and/or quality |

**Relevance, feasibility, and usefulness assessment**

Definitions for relevance, feasibility and usefulness were drawn from those used in the Global Action for Measurement of Adolescent Health (GAMA) indicator development process.^1^ The core group, measures and MHH expert group and exemplar country stakeholders rated indicators on the ‘PowerNoodle’ online web platform according to these criteria.

Relevance: The indicator measures a specific construct in a priority area of interest and there is a clear, demonstrated relationship between the indicator and menstrual health.

Feasibility: Data for the indicator can be obtained with reasonable and affordable effort.

Usefulness: The indicator captures information that is easily understood and timely. The information is easy to communicate to stakeholders and facilitates investment and action in menstrual health strategies, priorities, or programming.

^1^ GAMA Advisory Group. Proposed indicators for global adolescent health measurement by the Global Action for Measurement of Adolescent health (GAMA) Advisory Group. Version 20 October 2020. Available from https://www.who.int/docs/default-source/mca-documents/advisory-groups/gama/gama-list-of-indicators-draft-2-v20201020.pdf?sfvrsn=f6d00176_6 (last accessed October, 2022)]
